# Stigma and its associations with self-confidence and sexual relations in 4 types of premature ejaculation

**DOI:** 10.1186/s12610-024-00226-1

**Published:** 2024-07-02

**Authors:** Jishuang Liu, Tong Bao, Qunfeng Wang, Hui Jiang, Xiansheng Zhang

**Affiliations:** 1 Department of Urology, Anhui Province Key Laboratory of Genitourinary Diseases, The Institute of Urology, The first affiliated hospital of Anhui Medical University, Hefei, Anhui China; 2 Department of Urology, Anqing Municipal Hospital, Anqing Medical Center of Anhui Medical University, Renmin Road No. 352, Yingjiang District, Anqing, 246003 Anhui People’s Republic of China; 3 Department of Urology, Peking University First Hospital Institute of Urology, Peking University Andrology Center, Peking University First Hospital, No. 8 Xishiku Street Xicheng District, Beijing, China; 4 Department of Urology, The First Affiliated Hospital of Anhui Medical University, Hefei, China

**Keywords:** Éjaculation précoce, Stigmatisation, Confiance en soi, Relations sexuelles, Premature ejaculation, Stigma, Self-confidence, Sexual relationships

## Abstract

**Background:**

Although men with premature ejaculation (PE) always show more negative emotions, including embarrassment, guilt and worry, this may be related to the stigma of PE. To investigated stigma and its associations with self-confidence and sexual relations in 4 PE syndromes, a survey was conducted in our hospital from December 2018 to December 2019 among 350 men with self-reported PE and 252 men without self-reported PE. The stigma, self-confidence and sexual relations were assessed by the Social Impact Scale (SIS) and Self-Esteem and Relationship questionnaire (SEAR), respectively. Ejaculation control, sexual life satisfaction and distress caused by PE were evaluated by the Index of PE.

**Results:**

Men with self-reported PE had higher internalized shame and social isolation scores and lower SEAR scores than control subjects. The highest score of internalized shame and social isolation and the lowest score of SEAR appeared in men with lifelong PE (LPE). After age adjustment, the positive relationships were stronger between distress about PE and internalized shame. Whereas, the stronger negative associations were found between social isolation and sexual satisfaction. The strongest association was observed between social isolation and sexual relationship. Therefore, the stigma associated with PE adversely affects the self-confidence, self-esteem, and sexual relationships of men with PE.

**Conclusion:**

Men with PE, especially LPE, have a high level of stigma and disharmonious sexual relations, and often lack self-confidence and self-esteem, which have a certain negative impact on their physical and mental health and life. These will be the key issues to be considered when we formulate a personalized treatment plan for PE.

## Résumé

**Contexte:**

Bien que les hommes atteints d’éjaculation précoce (EP) montrent plus d’émotions négatives toujours, notamment de l’embarras, de la culpabilité et de l’inquiétude, cela peut être lié à la stigmatisation de l’EP. Afin d’étudier la stigmatisation et ses associations avec la confiance en soi et les relations sexuelles dans 4 syndromes d’EP, une enquête a été menée dans notre hôpital de décembre 2018 à décembre 2019 auprès de 350 hommes atteints d’EP autodéclarée et de 252 hommes sans EP autodéclarée. La stigmatisation, la confiance en soi et les relations sexuelles ont été évaluées respectivement à l’aide de l’échelle d’impact social (SIS) et du questionnaire sur l’estime de soi et les relations (SEAR). Le contrôle de l’éjaculation, la satisfaction de la vie sexuelle et la détresse causée par l’EP ont été évalués par l’indice d’EP.

**Résultats:**

Les hommes ayant une EP autodéclarée avaient des scores de honte intériorisée et d’isolement social plus élevés, et des scores SEAR inférieurs, à ceux des sujets témoins. Le score le plus élevé de honte intériorisée et d’isolement social, et le score le plus bas de SEAR, sont apparus chez les hommes atteints d’EP à vie (EPL). Après ajustement sur l’âge, les relations positives étaient plus fortes entre la détresse due à l’EP et la honte intériorisée. Les associations négatives les plus fortes ont été trouvées entre l’isolement social et la satisfaction sexuelle. Par conséquent, la stigmatisation associée à l’EP affecte négativement la confiance en soi, l’estime de soi et les relations sexuelles des hommes atteints d’EP.

**Conclusion:**

Les hommes atteints d’EP, en particulier ceux atteints d’EPL, ont un niveau élevé de stigmatisation et de relations sexuelles disharmonieuses, et ils manquent souvent de confiance en soi et d’estime de soi; ce qui a un impact négatif certain sur leur santé physique et mentale, et sur leur vie. Ce seront les questions clés à prendre en compte lorsque nous formulerons un plan de traitement personnalisé pour l’EP.

## Introduction

In the past few decades, numbers of definitions have been formed, but the 3 critical criteria which are used to characterize this condition clinically are generally agreed, including (i) short intravaginal ejaculation time, (ii) inability or poor ejaculatory control, and (iii) negative personal consequences [1]. In 2014, the International Society for Sexual Medicine (ISSM) further defined the definitions of lifelong PE (LPE) and acquired PE (APE) based on the evidence-based medical basis [2]. In addition, two other PE subtypes proposed by Waldinger, variable PE (VPE) and subjective PE (PSE), which did not meet the SISM diagnostic criteria but were deemed useful in treating patients, were temporarily accepted by the SISM as two special subtypes. VPE is defined as the patient's symptoms of rapid ejaculation always appear intermittently, while SPE is defined as the patient's ejaculation time is normal but always thinks that the ejaculation is too fast [3]. The emergence of these two classifications solved the problem of patients who did not meet the diagnostic criteria for PE, but still complained of PE, and after statistical analysis, about 5% of adult men in the world suffer from PE [2, 4, 5]. Subsequently, this was confirmed by Serefoglu et al., in their study, they found that among 2593 subjects, 2.3%, 3.9%, 8.5% and 5.1% were classified as LPE, APE, VPE and SPE, respectively [6], and a similar distribution was found in China by Zhang et al. [7].

PE has been reported as having a negative impact on men's physical and mental health [8, 9]. However, few men with PE had sought treatment from a physician or therapist [10]. The results from Carson et al. showed that only 1% of men aged ≥ 40 reported that they had received treatment for PE [11]. Numerous factors are obstacles to PE treatment, some of which are related to the patient's inner resistance [12]. Patients may be reluctant to discuss their PE with a physician because of the stigma associated with PE [13]. Stigma is a psychological stress response, which manifests as a sense of shame due to illness [14]. As a concept in psychology, stigma exists in different kinds of diseases, such as mental illness and AIDS, which affect the quality of life of patients [15, 16]. Of course, PE is no exception. However, up to now, there is no quantitative study on the stigma of PE patients and its influence on the quality of life.

Furthermore, the previous studies have shown that PE is not only associated with increased personal and interpersonal distress, but also with an alteration in patients' self-confidence and their relationships with their partners [17, 18]. Self-confidence is a kind of consciousness and psychological state that actively and effectively expresses self-worth, self-esteem and self-understanding, and reflects the confidence of individuals in their ability to complete an activity successfully [19].

There are individual differences in self-confidence, which affect the psychology and behavior of individuals in learning, competition, employment and achievement to varying degrees, and the same is true for PE patients. The work of Karakeci et al. has highlighted the role played by sexual partners in PE problems, a role they think is possibly more important than in other male sexual dysfunctions [18].

Studies by Hartmann et al. [20] and Symonds et al. [21] indicated that PE was associated with an impairment of the experience of sexual relationships. Worry about partner dissatisfaction, as well as generalized insecurity and lack of self-esteem, appear to have become a mental burden for men with PE [20].Therefore, self-confidence, self-esteem and the quality of sexual relationships can be considered as important parameters to be taken into account in the understanding and treatment of PE problems [21, 22]. Therefore, self-confidence, self-esteem and sexual relations may play a vital role in accelerating or maintaining PE.

Although the importance of stigma, self-confidence and relationship with partners is obvious in the occurrence and development of PE, there is still a lack of systematic research on these in PE. In our clinical work, we found that men with PE have a higher level of stigma and lower self-confidence, and the relationship between the sexes is not so harmonious. This may be an important factor affecting the treatment of these PE patients. Additionally, our previous work had shown that the psychological problems of PE are different among the 4 subtypes of PE [23]. Therefore, this study aimed to investigate the role of stigma, self-confidence, and gender relations in PE and their associations in the new classification of the 4 subtypes of PE. At the same time, the relationship between these risk factors, such as stigma and social isolation, and their role in different types of premature ejaculation were explored. These can not only reflect the true psychological conditions of PE patients, but also provide reference for their psychological intervention and behavior correction, which can provide a theoretical basis for clinicians to make personalized treatment plans.

## Materials and Methods

### Subjects

**Fig. 1:**
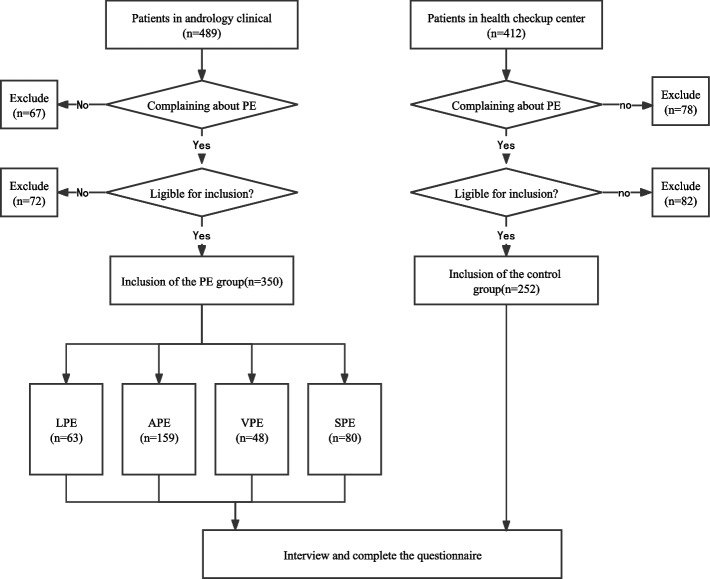
Flow chart of this study

From December 2018 to December 2019, a non-interventional, observational, cross-sectional field survey was conducted in The First Affiliated Hospital of Anhui Medical University. Subjects were recruited from men with self-reported PE who visited the andrology clinic of our hospital. Experienced andrologists assessed whether the men met the diagnostic criteria for specific types of PE, and if they met the criteria, they were included in the corresponding groups. With the deepening of the concept of early detection, early diagnosis and early treatment in preventive medicine, more and more people go to the physical examination center of the hospital for some routine screening projects to find out whether there are some diseases as early as possible without obvious discomfort. When these people were not in a disease state after routine examination and they were satisfied with their ejaculation control ability, they were included in the control group. Before subject enrollment, a careful medical and sexual history were recorded by an experienced clinician. Subjects who meet the following criteria can be included in this study: (a) male patients aged ≥ 18 years; (b) had a heterosexual, stable, and monogamous sexual relationship with the same female partner for at least 6 months; And (c) able to comprehend and speak Chinese. Men on medication that could have affected their ejaculatory function and /or psychological status were excluded (e.g., selective serotonin reuptake inhibitors). The specific flow chart is shown in Fig. 1.

Before the study enrollment, all subjects were informed of the study, and all the eligible men were required to provide written consent and were not allowed to be enrolled multiple times. In addition, to ensure that the contents of the questionnaire are comprehensive and easy to understand, a small number of subjects completed a prestudy (*N* = 30) to improve the originally designed items. We then calculated the required sample size using the following formula: n = [Z^2^*p*(1-p)]/e^2. This study was evaluated and approved by our University Research Subject Review Board.

### Study design

The survey was conducted through face-to-face interviews. Experienced clinicians should be assigned to carefully evaluate the medical and sexual history of each subject. Eligible subjects were asked to complete a verbal questionnaire, which included demographic information (e.g., age, body mass index [BMI], educational level, resident, lifestyle, and occupational status), medical and sexual history (e.g., self-estimated intravaginal ejaculation latency time [IELT]), and self-estimated scales [e.g., Social Impact Scale (SIS), Self-Esteem and Relationship Questionnaire (SEAR), Index of Premature Ejaculation(IPE)].The reliability of the instruments (SIS, SEAR and IPE) was assessed with Cronbach’s alpha coefficient ranged from 0.80 to 0.95 and was very good for all instruments [24, 25]. The flow of our study is shown in Fig. 1.

### Definition of PE

Men who were not satisfied with the time of ejaculation were accepted as having self-reported PE. Furthermore, each patient was divided into one of the following 4 categories: LPE, APE, VPE and SPE.

LPE is defined as premature ejaculation that occurs almost every time a patient has sexual intercourse; It almost always appears at the time of first sexual intercourse, persists throughout life (70%), and even worsens with age (30%), with a diminished or absent ability to delay ejaculation. APE refers to the occurrence of premature ejaculation at some point in a man's life, and the patient usually has a normal ejaculation experience before the first premature ejaculation. VPE is defined as inconsistent and irregular occurrence of early ejaculation, reduced or absent ability to delay imminent ejaculation, and reduced ability to ejaculate accompanied by short or normal ejaculation times. SPE is defined as a subjective feeling of consistent or inconsistent premature ejaculation during sexual intercourse, and there is a preoccupation with an imagined early ejaculation, or lack of ability to delay ejaculation; The actual intravaginal ejaculation latency time is in the normal range or may even be of longer duration.

### Measures

The following instruments were used to evaluate PE (IPE), stigma (SIS), self-confidence and sexual relations (SEAR).

- IPE: IPE is a 10-item reliable and valid questionnaire, which was developed as a measure to evaluate control over ejaculation (total of items 1,2,4,5), satisfaction with sex life (total of items 3,6,7,8) and distress (total of items 9,10) in men with PE [26]. The higher the IPE score, the stronger the ejaculation control, the higher the satisfaction of sexual life, and the less distress caused by PE. Additionally, IPE has been validated in Chinese [12].
- SIS: SIS was compiled by Fife in 2000, and was originally used to evaluate the stigma of cancer and AIDS patients [24]. In 2007, Pan et al. translated it into a Chinese version via a standardized two-stage translation procedure and applied it to patients with major depression, schizophrenia and AIDS, respectively. Studies have confirmed that the reliability and validity of the scale are satisfactory, and it can better evaluate patients' psychological problems. There are 24 items in the social impact scale, which are divided into 4 dimensions, including social rejection (9 items), financial insecurity (3 items), internalized shame (5 items) and social isolation (7 items). Social rejection reflects the discrimination felt in society and work; Financial insecurity, which is related to job stability and income, stems from the influence of discrimination on self-perception and interpersonal relationship; Internalized shame shows the internalization of social exclusion and economic insecurity, which leads to the need to hide one's illness from others. Social isolation refers to the feeling of loneliness, imbalance and uselessness with others. Social rejection and financial insecurity represent perceived shame and discrimination, while internalized shame and social isolation represent self-shame and discrimination. Regarding the answers to all items of the social impact scale, the Likert-style 4-point system is adopted, in which 4 points mean " strongly agree " and 1-point means " strongly disagree ". The total score of the scale is the sum of the scores of the 4 dimensions and a greater level of perceived stigmatization scored higher. Scores ranged from 24 to 96, with higher scores indicating more severe stigma. The correlations between these subscales ranged from 0.28 to 0.66 [27].
- SEAR: SEAR is a 14-item, self-administered questionnaire, which is only completed by men during the 4-week recall period. This questionnaire includes two dimensions, sexual relationship (items 1 to 8) and confidence (items 9 to 14), which produce a total score. The other two sub-dimensions are obtained from confidence, including self-esteem (items 9 to 12) and overall relationship (items 13 to 14). There are no cutoff values for SEAR, but its association with sexual dysfunction has been widely demonstrated. The higher the score, the better the functioning [25].The Chinese version of SEAR was widely used in the previous studies in China [28].

### Statistical analysis

All statistical analyses were performed using SPSS version 20.0(SPSS Inc, Chicago, IL, USA). Descriptive statistics were used to summarize the subjects’ characteristics. Data were expressed as mean ± standard deviation or number (percentage) when appropriate. Chi-squared test was used to compare categorical data. The independent *t*—test or analysis of variance was used to compare numerical data. The difference among the 4 subtypes of PE was measured by one-way analysis of variance. Associations between SIS, SEAR and IPE scores were assessed using partial correlations analysis. Ages ranged from 20 to 66 years in men with complaints of PE and as PE might be associated with age [12], all the data were adjusted for the age when the relationships between SIS, SEAR and IPE scores were measured. For all tests, *P* < 0.05 was considered statistically significant.

## Results

### Demographic information of the enrolled men

Finally, a total of 602 men (350 men with self-reported PE and 252 men without self-reported PE) were enrolled and completed the survey, with a response rate of 75.68%. The proportion of the 4 types of PE in men with self-reported PE was as follows: LPE, 18.00% (63/350); APE, 45.43% (159/350); VPE, 13.71% (48/350); SPE, 22.86% (80/ 350). Detailed demographic information for men with and without self-reported PE are summarized in Table 1. Similarly, we compared the detailed demographic information of all subjects in 4 types of PE (Table 2). Additionally, the etiological distribution of men with/ without self-reported PE and the 4 subtypes of PE are displayed in Table 3.

**Table 1:** Demographic information in men with and without self-reported PE

Characteristics	All (*n* = 602)	With PE (*n* = 350)	Without PE (*n* = 252)	*P* value
Age, years	38.39 ± 9.91	40.63 ± 10.74	35.29 ± 8.76	< 0.001
BMI, kg/m2	24.45 ± 3.63	24.73 ± 3.91	24.06 ± 3.25	0.46
Self-estimated IELT, minutes	2.86 ± 1.36	2.20 ± 1.15	3.78 ± 1.64	< 0.001
Duration of the relationship, years	24.53 ± 12.6	23.67 ± 11.24	25.73 ± 14.49	0.54
Frequency of sexual intercourse in the past 4 weeks, times	5.55 ± 2.62	5.02 ± 2.95	6.29 ± 2.17	< 0.001
Lifestyle, n (%)				
Smoking	359(59.63)	227 (64.86)	132 (52.38)	< 0.001
Exercise	309(51.33)	150 (42.86)	159 (63.10)	< 0.001
Educational status, n (%)				0.30
High school or less	209(34.72)	115 (32.86)	94 (37.30)	
University graduate	393(65.28)	235 (67.14)	158 (62.70)	
Occupational status, n (%)				0.34
Student	168(27.91)	92 (26.29)	76 (30.16)	
Unemployed	149(24.75)	90 (25.71)	59 (23.41)	
Employed	285(47.34)	168 (48.00)	117 (46.43)	
Resident, n (%)				0.31
Urban	271(45.02)	151 (43.14)	120 (47.62)	
Rural	331(54.98)	199 (56.86)	132 (52.38)	

Data are expressed as mean ± SD or number (percentage), as appropriate

Differences between men with and without PE were assessed by independent t-test or chi-square test as appropriate

*P* value: differences between men with and without PE

*PE* Premature ejaculation, *BMI* Body mass index, *IELT* intravaginal ejaculation time

**Table 2:** Demographic information in men with different PE syndromes

Characteristics	LPE ^*^ (*n* = 63)	APE^*^ (*n* = 159)	VPE^*^ (*n* = 48)	SPE^*^ (*n* = 80)	*P* value^†^
Age, years	38.72 ± 11.75^b,c,d^	46.53 ± 11.72^a,c,d^	30.09 ± 8.94^a,b,d^	36.72 ± 9.02^a,b,c^	< 0.001
BMI, kg/m2	22.85 ± 3.92	26.46 ± 3.84	23.76 ± 4.02	23.35 ± 3.83	0.38
Self-estimated IELT, minutes	1.25 ± 0.75^b,c,d^	1.76 ± 0.91^a,c,d^	2.81 ± 1.44^a,b,d^	3.45 ± 1.29^a,b,c^	< 0.001
Duration of the relationship, years	23.35 ± 10.72	20.08 ± 7.57	26.87 ± 14.45	29.15 ± 11.04	0.64
Frequency of sexual intercourse in the past 4 weeks, times	4.45 ± 2.36^c,d^	5.02 ± 2.54^c,d^	6.57 ± 3.82^a,b^	4.55 ± 2.75^a,b^	< 0.001
Lifestyle, n (%)					
Smoking	34 (53.97)^b^	131 (82.39)^a,c,d^	24 (50.00)^b^	38 (47.50)^b^	< 0.001
Exercise	26 (41.27)^c^	57 (35.85)^c,d^	26 (54.17)^a,b^	41 (51.25)^b^	< 0.001
Educational status, n (%)					
High school or less	18 (28.57)	50 (31.45)	19 (39.58)	28 (35.00)	0.61
University graduate	45 (71.43)	109 (68.55)	29 (60.42)	52 (65.00)	0.61
Occupational status, n (%)					
Student	13 (20.63)	45 (28.30)	11 (22.92)	23 (28.75)	0.59
Unemployed	15 (23.81)	41 (25.79)	13 (27.08)	21 (26.25)	0.98
Employed	35 (55.56)	73 (45.91)	24 (50.00)	36 (45.00)	0.56
Resident, n (%)					
Urban	27 (42.86)	72 (45.28)	19 (39.58)	33 (41.25)	0.45
Rural	36 (57.14)	87 (54.72)	29 (60.42)	47 (58.75)	0.88

*PE* Premature ejaculation, *APE* Acquired PE, *BMI* Body mass index, *IELT* intravaginal ejaculation time, *LPE* Lifelong PE, *SPE* Subjective PE, *VPE* Variable PE

Data are expressed as mean ± SD or number (percentage), as appropriate

^*^Difference between two subgroups assessed by *t*-test or chi-square test, as appropriate

^†^Difference among 4 PE syndromes assessed by one-way analysis of variance or chi-square test, as appropriate

^a^Significant difference compared with LPE

^b^Significant difference compared with APE

^c^Significant difference compared with VPE

^d^Significant difference compared with SPE

**Table 3:** Etiology of PE includes the 4 types of PE

Etiology	All (*n* = 602)	With PE (*n* = 350)	Without PE (*n* = 252)	*P* value^*&*^	LPE ^*^ (*n* = 63)	APE^*^ (*n* = 159)	VPE^*^ (*n* = 48)	SPE^*^ (*n* = 80)	*P* value^†^
Anxiety	164(27.24)	134(22.26)	30(11.9)	< 0.001	18 (28.57%)^k,{^	65 (40.88%)^k,{^	13 (27.08%)^‡,§^	38 (47.50%)^‡,§^	< 0.05
Depression	117(19.44)	101(16.78)	16(6.35)	< 0.001	12 (19.05%)^{^	55 (34.59%)^{^	8 (16.67%)^{^	26 (32.50%)^‡,§,^^*k*^	< 0.05
Sexual desire disorder	131(21.76)	102(16.94)	29(11.51)	< 0.001	13 (20.63%)^§,k,{^	73 (45.91%)^‡,^^*k*^^,^^*{*^	6 (12.50%)^‡,§^	10 (12.50%)^‡,^^*{*^	< 0.05
Hypertension	64(25.40)	50(8.31)	14(5.56)	0.001	6 (9.52%)^§^	32 (20.13%)^‡,^^*k*^^,^^*{*^	4 (8.33%)^§^	8 (10.00%)^§^	< 0.05
Diabetes mellitus	36(5.98)	30(4.98)	6(2.38)	0.002	3 (4.76%)^§^	21 (13.21%)^‡,^^*k*^^,^^*{*^	2 (4.17%)^§^	4 (5.00%)^§^	< 0.05
Varicocele	70(11.63)	47(7.81)	23(9.13)	0.104	8 (12.70%)	23 (14.47%)	6 (12.50%)	10 (12.50%)	0.965
CP	209(34.72)	160(26.58)	49(19.44)	< 0.001	19 (30.16%)^§^	96 (60.38%)^‡,^^*k*^^,^^*{*^	14 (29.17%)^§,{^	31 (38.75%)^‡,§,^^*k*^	< 0.05
ED	127(21.1)	99(16.45)	28(11.11)	< 0.001	16 (25.40%)^§,k,{^	63 (39.62%)^‡,^^*k*^^,^^*{*^	6 (12.50%)^‡,§,^^*{*^	14 (17.50%)^‡,§,^^*k*^	< 0.05

*PE* Premature ejaculation, *APE* acquired PE, *LPE* lifelong PE, *VPE* variable PE, *SPE* Subjective PE, *CP* Chronic prostatitis, *ED* Erectile dysfunction

Data are expressed as number (percentage)

^*&*^Difference between men with and without self-reported PE assessed by t-test or chi-square test, as appropriate

^*^Difference between 2 subgroups assessed by one-way analysis of variance or chi-square test, as appropriate

^†^Difference among 4 types of PE assessed by one-way analysis of variance or chi-square test, as appropriate

^‡^Significant difference compared with LPE

^§^Significant difference compared with APE

^*k*^ Significant difference compared with VPE

^*{*^Significant difference compared with SPE

### Outcomes of SIS and SEAR scores in men with and without self-reported PE

As shown in Table 4, there were significant differences between men with and without self-reported PE with regard to the total scores of SIS and SEAR. The total scores of SIS were found to be significantly higher in men with self-reported PE, whereas the total SEAR scores were significantly lower (*P* < 0.001 for all). As for the 4 dimensions of SIS, the significant differences were only seen in the two of internalized shame and social isolation, and the group with self-reported PE have higher scores (*P* < 0.001 for all). Similarly, the two dimensions of SEAR have statistical differences between the 2 groups, and the statistical difference also applies to the two sub-dimensions of the self-confidence dimension, including self-esteem and overall relationship (*P* < 0.001 for all). Unlike SIS, the group with self-reported PE have lower scores (*P* < 0.001 for all).

**Table 4:** Outcomes of SIS, SEAR and IPE in all subjects

Inventory	With PE (*n* = 350)	Without PE (*n* = 252)	*P* value	LPE (*n* = 63)	APE (*n* = 159)	VPE (*n* = 48)	SPE (*n* = 80)	*P* value
SIS:								
Total score	56.47 ± 14.38	49.28 ± 15.84	< *0.001*	59.49 ± 17.72 ^†,‡,§^	55.91 ± 14.47 ^*,‡,§^	52.38 ± 13.37 ^*,†,§^	54.86 ± 14.80 ^*,†,‡^	< *0.001*
Social rejection	19.96 ± 6.03	19.24 ± 6.69	0.56	20.84 ± 6.62	19.02 ± 6.20	20.96 ± 5.88	20.54 ± 5.92	0.64
Financial insecurity	6.83 ± 2.39	6.82 ± 3.02	0.48	6.99 ± 2.66	6.74 ± 3.21	6.89 ± 2.80	6.85 ± 2.86	0.61
Internalized shame	13.52 ± 3.10	12.29 ± 3.26	< *0.001*	15.64 ± 3.62 ^†,‡,§^	14.83 ± 3.20^*,‡,§^	9.07 ± 2.27^*,†,§^	11.92 ± 2.68 ^*,†,‡^	< *0.001*
Social isolation	15.52 ± 4.93	10.93 ± 4.36	< *0.001*	16.02 ± 4.52 ^†,‡,§^	15.32 ± 4.66^*,‡,§^	15.46 ± 4.47^*,†,§^	15.56 ± 4.60 ^*,†,‡^	< *0.001*
SEAR:								
Total score	61.28 ± 15.52	85.57 ± 10.19	< *0.001*	60.67 ± 15.80 ^†,‡,§^	60.89 ± 15.30^*,‡,§^	62.26 ± 16.62^*,†,§^	61.94 ± 16.04 ^*,†,‡^	< *0.001*
Sexual relationship	55.04 ± 17.74	80.69 ± 13.37	< *0.001*	54.02 ± 17.77 ^†,‡,§^	55.29 ± 16.48^*,‡,§^	55.33 ± 18.84^*,†,§^	55.16 ± 16.92 ^*,†,‡^	< *0.001*
Confidence:	71.43 ± 22.08	90.45 ± 15.27	< *0.001*	70.25 ± 22.29 ^†,‡,§^	71.73 ± 23.36^*,‡,§^	72.62 ± 23.37^*,†,§^	71.04 ± 21.19 ^*,†,‡^	< *0.001*
Self-esteem	73.54 ± 21.35	91.17 ± 12.25	< *0.001*	73.07 ± 19.88 ^†,‡,§^	73.28 ± 22.29^*,‡,§^	74.47 ± 20.64^*,†,§^	73.85 ± 19.94 ^*,†,‡^	< *0.001*
Overall relationship	69.02 ± 20.27	88.62 ± 18.84	< *0.001*	68.39 ± 20.33 ^†,‡,§^	68.84 ± 21.2^*,‡,§^5	69.33 ± 19.96^*,†,§^	69.69 ± 22.05 ^*,†,‡^	< *0.001*
Index of PE								
Total score (Q1-Q10)	21.31 ± 9.75			21.73 ± 9.98	13.84 ± 5.82	28.83 ± 11.28	31.31 ± 13.45	
Sexual satisfaction (Q3, Q6, Q7, Q8)	11.26 ± 4.07			10.74 ± 3.73	8.29 ± 2.92	14.68 ± 5.85	15.52 ± 6.47	
Control over ejaculation (Q1, Q2, Q4, Q5)	5.93 ± 2.65			6.42 ± 2.88	3.27 ± 1.80	8.64 ± 3.47	9.20 ± 4.26	
Distress about PE (Q9, Q10)	4.12 ± 1.69			4.57 ± 2.03	2.28 ± 1.09	5.51 ± 2.02	6.59 ± 3.37	

*PE* Premature ejaculation, *APE* Acquired PE, *LPE* Lifelong PE, *VPE* variable PE, *SPE* subjective PE, *SIS* Social Impact Scale, *SEAR* Self-Esteem and Relationship questionnaire

Data are expressed as mean ± SD or number (percentage), as appropriate

Differences between men with and without PE were assessed by independent t-test as appropriate

Difference among 4 PE syndromes was assessed by one-way analysis of variance

*P* value: differences between men with and without PE

^*^Significant difference compared with LPE

^†^Significant difference compared with APE

^‡^Significant difference compared with VPE

^§^Significant difference compared with SPE

### Outcomes of SIS, SEAR and IPE scores in men with the 4 PE Syndromes

As shown in Table 4, the significant differences observed among men with different types of PE not only in SIS, but also in SEAR (*P* < 0.001 for all). For the total scores of SIS, they were higher in men with LPE (59.49 ± 17.72), whereas lower in VPE (52.38 ± 13.37). As for the two dimensions of internalized shame and social isolation, higher scores appeared in men with LPE (Internalized shame: 15.64 ± 3.62; Social isolation: 16.02 ± 4.52). However, the lower scores were different, the lowers were appeared in men with VPE and APE, respectively (Internalized shame: 9.07 ± 2.27; Social isolation: 15.32 ± 4.66).

Regarding SEAR, the total scores were higher in men with VPE (62.26 ± 16.62) and lower in men with LPE (60.67 ± 15.80). Concerning the two dimensions of SEAR (sexual relationship and confidence), the higher scores and the lower scores were appeared in men with VPE and LPE, respectively (Sexual relationship: 55.33 ± 18.84 vs. 54.02 ± 17.77; Confidence: 72.62 ± 23.37 vs. 70.25 ± 22.29). What is different is the two sub-dimensions of confidence (self-esteem and overall relationship), lower values were all observed in men with LPE (73.07 ± 19.88 vs 68.39 ± 20.33), while the higher in self-esteem men were seen in men with VPE (74.47 ± 20.64) and in overall relationship were in men with SPE (69.69 ± 22.05). Additionally, for the total and subdomain of IPE, men with SPE scored higher than those other subtypes of PE (*P* < 0.001 for all).

### Interrelationship between internalized shame, social isolation, SEAR and IPE scores in men with self-reported PE

Association between internalized shame, social isolation, SAER and IPE scores were observed in men with self-reported PE (Table 5). After age adjustment, internalized shame scores were positively associated with total and subdomain scores of IPE (distress about PE), while the negative associations were found in the items of sexual satisfaction and control over ejaculation (*P* < 0.001 for all). Similar relationships between social isolation score and total and subdomain scores of IPE were also found (*P* < 0.001 for all). These correlations happened to be the opposite in the comparison of SEAR and IPE scores. The negative correlations were seen between SEAR and the total and subdomain scores of IPE (distress about PE), while the positive associations were found in the items of sexual satisfaction and control over ejaculation (*P* < 0.001 for all). Additionally, for internalized shame, the stronger relationships were observed in the subdomain of distress about PE (adjusted r = 0.72, *P* < 0.001). And for social isolation and SEAR, the stronger associations were seen in the subdomain of sexual satisfaction (social isolation: adjusted r = -0.50, *P* < 0.001; sexual relationship: adjusted r = 0.56, *P* < 0.001; confidence: adjusted r = 0.47, *P* < 0.001; self-esteem: adjusted r = 0.47, *P* < 0.001; overall relationship: adjusted r = 0.53, *P* < 0.001;).

**Table 5:** Relationships between SIS, SEAR and IPE in men with self-reported PE

	IPE							
	Total scores		Sexual satisfaction		Control over ejaculation		Distress about PE	
	Adjusted r	*P*	Adjusted r	*P*	Adjusted r	*P*	Adjusted r	*P*
SIS:								
Internalized shame	0.66	< 0.001	-0.54	< 0.001	-0.33	< 0.001	0.72	< 0.001
Social isolation	0.44	< 0.001	-0.50	< 0.001	-0.36	< 0.001	0.39	< 0.001
SEAR:								
Sexual relationship	-0.46	< 0.001	0.56	< 0.001	0.39	< 0.001	-0.40	< 0.001
Confidence:	-0.4	< 0.001	0.47	< 0.001	0.35	< 0.001	-0.45	< 0.001
Self-esteem	-0.43	< 0.001	0.47	< 0.001	0.40	< 0.001	-0.42	< 0.001
Overall relationship	-0.5	< 0.001	0.53	< 0.001	0.37	< 0.001	-0.41	< 0.001

Correlations between outcomes of the SIS, SEAR and IPE scores were assessed using partial correlations

*PE* premature ejaculation, *IPE* Index of PE, *SIS* Social Impact Scale, *SEAR* Self-Esteem and Relationship questionnaire

### Association between internalized shame, social isolation and SEAR scores in men with different types of PE syndromes

To further understand the effect of stigma on PE, we further analyzed the correlation between internalized shame, social isolation and SERA scores in 4 types of PE syndromes. As shown in Table 6, we found that internalized shame and social isolation were influenced by each other in all the dimensions of SEAR in the 4 types of PE. Moreover, stronger relationships were observed in men with LPE. As for internalized shame, it has the strongest correlation with self-confidence and its sub-dimensions: self-esteem and overall relationship (Confidence: adjusted r = -0.63, *P* < 0.001; Self-esteem: adjusted r = -0.59, *P* < 0.001; Overall relationship: adjusted r = -0.55, *P* < 0.001). Similarly, the strongest association was observed between social isolation and sexual relationship (adjusted r = -0.57, *P* < 0.001).

**Table 6:** Association between internalized shame, social isolation and SEAR scores in men with different types of PE syndromes

	SEAR	Sexual relationship		Confidence		Self-esteem		Overall relationship	
PE syndromes	SIS	Adjusted r	*P*	Adjusted r	*P*	Adjusted r	*P*	Adjusted r	*P*
PE	Internalized shame	-0.51	< 0.001	-0.53	< 0.001	-0.49	< 0.001	-0.50	< 0.001
	Social isolation	-0.43	< 0.001	-0.45	< 0.001	-0.42	< 0.001	-0.40	< 0.001
LPE	Internalized shame	-0.48	< 0.001	-0.63	< 0.001	-0.59	< 0.001	-0.55	< 0.001
	Social isolation	-0.57	< 0.001	-0.55	< 0.001	-0.52	< 0.001	-0.49	< 0.001
APE	Internalized shame	-0.52	< 0.001	-0.49	< 0.001	-0.46	< 0.001	-0.43	< 0.001
	Social isolation	-0.47	< 0.001	-0.44	< 0.001	-0.41	< 0.001	-0.42	< 0.001
VPE	Internalized shame	-0.37	< 0.001	-0.36	< 0.001	-0.31	< 0.001	-0.32	< 0.001
	Social isolation	-0.40	< 0.001	-0.45	< 0.001	-0.35	< 0.001	-0.37	< 0.001
SPE	Internalized shame	-0.51	< 0.001	-0.60	< 0.001	-0.39	< 0.001	-0.42	< 0.001
	Social isolation	-0.42	< 0.001	-0.44	< 0.001	-0.40	< 0.001	-0.36	< 0.001

Correlations between outcomes of the internalized shame, social isolation and SEAR scores were assessed using partial correlations

*PE* Premature ejaculation, *APE* Acquired PE, *LPE* Lifelong PE, *VPE* Variable PE, *SPE* subjective PE, *SIS* Social Impact Scale, *SEAR* Self-Esteem and Relationship questionnaire

## Discussion

To the best of our knowledge, this is the first cross-sectional study to systematically assess the stigma, self-confidence, relationship with partners and their associations in men with self-reported PE, especially in the new classification of 4 subtypes of PE. From the previous studies, we know that the 4 types of PE provide a new perspective for better understanding of PE [4]. Based on the new classification, we investigated the stigma of men with self-reported PE using SIS, which is specially developed for stigma research, and the role of self-confidence and gender relationship in PE through SEAR. These can help us understanding the psychological problems of PE from another unique perspective, which is of great significance for personalized treatment for PE.

In the current study, we showed that the scores of internalized shame and social isolation were significantly higher in men with self-reported PE (*P* < 0.001). That is to say, men with self-reported PE have a strong sense of subjective self-shame and social isolation than those without PE. This is in accordance with the context of a psychophysiological analysis, men with PE consistently showed greater negative emotion – including worry, embarrassment, guilt, and annoyance [29, 30].

Internalized shame is the result of experiencing isolation and rejection, including the feeling of separation from others, blaming yourself for illness and the need for confidentiality [24]. It is characterized by blame or depreciation, which is based on the experience, perception, or reasonable expectation of negative social judgments of health problems. Due to fear of being stigmatized by others, people with internalized shame may act in a way that is not conducive to dealing with diseases, such as keeping their diseases in secrecy and not seeking medical help when needed [31]. In the present study, we found that the scores of internalized shame in men with self-reported PE were significantly higher than those without self-reported PE. This means that men with PE have a strong sense of internal shame, which has become a stumbling block for them to seek help [32]. This is consistent with the analysis results from the Global Study of Sexual Attitudes and Behaviors (GSSAB), which demonstrated that a majority (77.8%) of men did not consult a doctor or other healthcare provider about their sexuality, and only 18.0% of participants sought medical help [33].

Social isolation means a sense of anomie in the traditional sociological sense, including loneliness, inequality with others and uselessness [24]. Previous studies have shown that perceived social isolation activates nerve, neuroendocrine and behavioral responses that promote short-term self-protection and has a destructive effect on human health [32, 34]. These neurological and behavioral influences include the increase of implicit vigilance against social threats, as well as the increase in anxiety, hostility and social withdrawal; reduce impulse control, increase negative emotions and depressive symptoms [35]. Furthermore, in many previous studies, the correlation between PE and negative emotions (depression or anxiety) has also been proved [36, 37]. This means that men with social isolation are more prone to have PE. Consistent with this, men with self-reported PE had higher scores of social isolations than those without self-reported PE in our study.

Additionally, among the 4 types of PE, the highest score of internalized shame and social isolation appeared in men with LPE. In others word, men with LPE have a higher level of stigma compared with other types of PE. This may be related to the sexual experience of patients with LPE. Men with LPE ejaculate within seconds or a minute since their first sexual experiences, with every partner, and throughout their life [38]. Such a long-term failed sexual experience makes them feel humiliated and many patients even think that PE is a shame and their own fault, so that they are unwilling to talk about PE with family, friends or doctors. It is precisely because of the stigma of PE that many PE patients cannot get better medical treatment, and even some patients with PE have given up treatment by themselves, which has a great impact on their lives and those of their partners.

For the other three types of PE patients, it is possible that they congregate normal sexual experiences they have had in the past with unfavorable experiences during the interview, resulting in better performance on social isolation and shame. Although a large number of studies have systematically investigated the negative relationship between stigma and willingness to seek medical treatment for other diseases (such as mental illness and dementia), there is no research on PE stigma [39]. Studies have shown that 50% of patients with heart disease want their doctors to be interested in their sexual health, but only 12.5% of cases focus on this topic. Our research is the first systematic study on stigma of PE, especially in the 4 types of PE, so that we can better understand the psychology of PE patients and provide them with better psychological intervention and behavior correction.

To better understand the influence of stigma on PE, we further investigated the self-confidence and sexual relations of men with self-reported PE. In our study, our results showed that not only the total scores of SEAR, but also the scores of all dimensions were significantly lower in men with self-reported PE than those without self-reported PE, including sexual relationship, confidence, self-esteem, and overall relationship. According to the guidelines on PE issued by the European Association of Urology, a necessary factor for premature ejaculation is a state of anxiety and depression secondary to rapid ejaculation. Previous studies have shown that anxiety and depression have a significant impact on subjects' self-confidence. And poor ejaculation control leads to poor sexual performance, which will also lead to poor sexual relationships. It can be seen from these that men with PE cannot satisfy their sexual partners, which can easily lead to disharmony between the two sexes. And PE itself also damage the patient's self-esteem and self-confidence [8]. If the partner cannot understand the patient at this time, or even complain about the patient, it will often cause severe damage to the self-esteem and self-confidence of the PE patient, and further aggravate PE. These fall into a vicious circle. This is consistent with previous research results [39–42].

Additionally, it is worth noting that among the 4 types of PE, the scores of LPE, including all dimensions, are the lowest. That is to say, among the 4 types of PE, men with LPE have the worst confidence and sexual relationship. Further analyzed the relationship between internalized shame, social isolation and SEAR scores, we found that internalized shame and social isolation were negatively correlated with all dimensions of SEAR in men with self-reported PE. The strongest relationships were observed in men with LPE. For internalized shame, it has the strongest correlation with self-confidence and its sub-dimensions: self-esteem and overall relationship. Similarly, the strongest association was observed between social isolation and sexual relationship. The potential reason may be that, as we mentioned above, the long-term failed sexual behavior of patients with LPE makes them physically and mentally exhausted.

On the IPE scale, we found that men with SPE scored highest in the total and subdomain scores; However, the lowest appeared in men with APE. The difference between APE and other three types of PE is that men with APE usually have PE due to the environment at some point in their life, and APE is usually more related to the underlying medical cause, such as ED and prostatitis [43]. As for SPE, these people often have normal or prolonged intercourse latency, but they still think they have PE. This is not a real disease or pathological state, but a combination of the patients’ understanding of PE, social and psychological factors, and sexual partners [44]. In addition, the associations between SIS, SEAR and IPE scores in men with self-reported PE were obviously. Based on the adjusted r values, the positive relationships were stronger between the distress about PE and internalized shame; Whereas the stronger negative associations were found between social isolation and sexual satisfaction (*P* < 0.001 for all). In other words, the stronger the stigma (internalized shame and social isolation), the more distressed about PE and the worse the sexual satisfaction. This is accordance with the study conducted by Hughes et al., in their study, they found that internalized shame was related to higher emotional distress and was an essential part of emotional distress [45]. And also, the correlation between loneliness and sexual life-satisfaction had been proved by Buczak-Stec et al. [46]. Regarding SEAR, positive correlations were stronger in sexual satisfaction and all domains of SEAR(*P* < 0.001 for all). That is to say, the more harmonious sexual relations, the stronger self-esteem and self-confidence, and the better sexual satisfaction. This means that psychological relationship factors play a critical role in subjective feelings of sexual function. Our findings are consistent with other reports emphasizing the importance of the sexual relationship in determining sexual satisfaction [47].

### Limitation of the study

However, we must consider that the current research has certain limitations. First, the cross-sectional design limits the research on stigma and its related influencing factors. It may be feasible to investigate through longitudinal follow-up studies; Second, many other factors may affect the stigma associated with PE, such as personality characteristics, religious beliefs, etc. Future research should examine factors related to the stigma associated with PE in a broader range of variables. Third, the current sample does not represent the entire population of PE and it will inevitably bias the results. Fourth, the main limitation is that the universality of this research may be limited by a single cultural/social background. The participants of this study mainly come from Chinese urban samples, and the local culture will inevitably have an impact on this study. Therefore, more research needs to be conducted in more countries and regions.

## Conclusion

This study provides evidence on stigma and its associations with self-confidence and sexual relations in men with self-reported PE, suggesting that these patients are the target population for stigma interventions. Importantly, our research provides empirical clues for developing public intervention strategies to reduce the stigma of male PE patients. The disharmony of sexual life and the decline of the overall relationship between the sexes in turn aggravate the stigma of PE, leading to the deterioration of self-confidence and self-esteem, thus aggravating PE and making it more difficult to treat PE. Therefore, it is necessary to treat the problem of PE correctly, carefully analyze the psychological state of men with PE, patiently conduct psychological counseling and reduce the stigma of PE to enable patients to establish self-confidence, obtain sexual pleasure, and get rid of bad psychological state, which is of great significance to the treatment of PE.

## Data Availability

The data used during the current study are available from the corresponding author on reasonable request.

## Abbreviations

APE: Acquired PE
BMI: Body mass index
GSSAB: The Global Study of Sexual Attitudes and Behaviors
IELT: Intravaginal ejaculation latency time
IPE: Index of Premature Ejaculation
ISSM: The International Society for Sexual Medicine
LPE: Lifelong PE
PE: Premature ejaculation
SEAR: Self-Esteem and Relationship Questionnaire
SIS: The Social Impact Scale
SPE: Subjective PE
VPE: Variable PE
