# Correction: Stigma and its associations with self-confidence and sexual relations in 4 types of premature ejaculation

**DOI:** 10.1186/s12610-024-00246-x

**Published:** 2024-12-09

**Authors:** Jishuang Liu, Tong Bao, Qunfeng Wang, Hui Jiang, Xiansheng Zhang

**Affiliations:** 1 Department of Urology, Anhui Province Key Laboratory of Genitourinary Diseases, The Institute of Urology, The first affiliated hospital of Anhui Medical University, Hefei, Anhui China; 2 Department of Urology, Anqing Municipal Hospital, Anqing Medical Center of Anhui Medical University, Renmin Road No. 352, Yingjiang District, Anqing, 246003 Anhui People’s Republic of China; 3 Department of Urology, Peking University First Hospital Institute of Urology, Peking University Andrology Center, Peking University First Hospital, No. 8 Xishiku Street Xicheng District, Beijing, China; 4 Department of Urology, The First Affiliated Hospital of Anhui Medical University, Hefei, China

**Correction** **:** **Basic Clin Androl 34, 11 (2024)**

**https://doi.org/10.1186/s12610-024-00226-1**

Following publication of the original article [1], the authors reported an error in affiliation 1. The incorrect and correct affiliation are given below:

Incorrect affiliation 1

Department of Urology, Anhui Province Key Laboratory of Genitourinary Diseases, The Institute of Urology, The First Affiliated Hospital of Anhui Medical UniversityAnhui Medical University, Hefei, Anhui, China.

Correct affiliation 1

Department of Urology, Anhui Province Key Laboratory of Genitourinary Diseases, The Institute of Urology, The first affiliated hospital of Anhui Medical University, Hefei, Anhui, China.

The original article [1] has been corrected.

## References

[1] Liu J, Bao T, Wang Q, et al. Stigma and its associations with self-confidence and sexual relations in 4 types of premature ejaculation. Basic Clin Androl. 2024;34:11. 10.1186/s12610-024-00226-1. 38951750 10.1186/s12610-024-00226-1 PMC11218248

